# Comparison of hospital-wide and age and location - stratified antibiograms of *S. aureus, E. coli,* and *S. pneumoniae*: age- and location-stratified antibiograms

**DOI:** 10.1186/2193-1801-2-63

**Published:** 2013-02-22

**Authors:** Sanjeev K Swami, Ritu Banerjee

**Affiliations:** 1 Division of Infectious Diseases, Nemours/Alfred I. duPont Hospital for Children, Wilmington, DE USA; 2 Department of Pediatric and Adolescent Medicine, Mayo Clinic, 200 First Street, SW, Rochester, MN 55905 USA

**Keywords:** Antibiogram, *S. aureus*, *E. coli*, *S. pneumoniae*, Age-stratified

## Abstract

**Background:**

Antibiograms created by aggregating hospital-wide susceptibility data from diverse patients can be misleading. To demonstrate the utility of age- and location-stratified antibiograms, we compared stratified antibiograms for three common bacterial pathogens, *E. coli, S. aureus*, and *S. pneumoniae*. We created stratified antibiograms based on patient age (<18 years, 18–64 years, >/=65 years), and inpatient or outpatient location using all 2009 *E. coli* and *S. aureus*, and all 2008–2009 *S. pneumoniae* isolates submitted to our clinical microbiology laboratory. We compared susceptibility rates among cumulative and stratified antibiograms using descriptive statistics.

**Findings:**

For *E. coli* and *S. aureus*, the institution-wide antibiogram overestimated resistance in pediatic isolates and underestimated resistance in isolates from the elderly. For *E. coli,* pediatric isolates were less susceptible to ampicillin and ampicillin-sulbactam and more susceptible to gentamicin and ciprofloxacin compared to adult isolates (p < 0.05 for all), and isolates from patients >65 years were least susceptible to ciprofloxacin (71%). For *S. aureus,* susceptibility to oxacillin, clindamycin, and levofloxacin was highest among children and decreased with increasing age (p < .001 for all). For *S. pneumoniae,* pediatric isolates were less susceptible than adult isolates to all agents except penicillin (IV breakpoint). Within children there were significant differences in susceptibility of inpatient and outpatient isolates of *E. coli* but not of *S. aureus* or *S. pneumoniae.*

**Conclusions:**

Stratified antibiograms reveal age - associated differences in susceptibility of *E. coli, S. aureus*, and *S. pneumoniae* that are obscured by hospital-wide antibiograms. Age-stratified antibiograms have potential to influence antibiotic selection.

## Introduction

Surveillance of local antimicrobial resistance is an integral part of antimicrobial stewardship. In many institutions, antimicrobial resistance rates are reported using hospital-wide, cumulative antibiograms. Cumulative antibiograms that aggregate data across a hospital can obscure differences among patient populations (e.g. children, adults, or long-term care facility residents), hospital units (e.g. outpatient, ward, nursery, intensive care unit), or anatomic sites (e.g. blood, respiratory, urine). Accordingly, the Clinical and Laboratory Standards Institute (CLSI) has published guidelines recommending stratification of susceptibility data by patient location (e.g. specific ward or long term care facility), clinical service, specimen type, or patient population (e.g. cystic fibrosis patients) (Clinical and Laboratory Standards Institute 2006; Hindler & Stelling 2007).

Despite publication of these guidelines and studies demonstrating the utility of stratified antibiograms (Binkley et al. 2006; Kaufman et al. 1998; Kuster et al. 2008), there continues to be great variability in antibiogram creation and reporting (Xu et al. 2012). Among 65 academic centers in the US, two thirds combined outpatient and inpatient isolates in their antibiograms, and half did not create unit-specific antibiograms (Lautenbach & Nachamkin 2006). It is not clear how many institutions create age group-specific antibiograms. A recent study reported that 6 of 10 pediatric hospitals within larger adult hospitals had cumulative antibiograms without stratification by age (Boggan et al. 2012).

To demonstrate the feasibility and utility of creating stratified antibiograms at our institution, we constructed and compared age and location -stratified antibiograms for three common bacterial pathogens, *E. coli, S. aureus*, and *S. pneumoniae*.

## Methods

Mayo Clinic, in Rochester, MN, is a tertiary care referral center for patients of all ages. Its children’s hospital is not a free-standing facility, but rather a hospital within the adult hospital. Mayo Clinic creates an institution- wide antibiogram that aggregates susceptibility data of isolates from inpatients and outpatients of all ages and specimen types. We created stratified antibiograms based on patient age (<18 years, 18–64 years, >/=65 years), and location of specimen collection (inpatient [IP] or outpatient [OP]) using all 2009 *E. coli* and *S. aureus*, and all 2008–2009 *S. pneumoniae* isolates. We excluded isolates that were not tested for antibiotic susceptibilities and included one isolate per patient per specimen source. Specimen sources were blood or respiratory (all pathogens), urine (*E. coli* and *S. aureus),* and soft tissue and bone (*S. aureus*). Susceptibility was defined using the 2009 CLSI breakpoints. Intermediate susceptibility was considered nonsusceptible. *X*^2^ or Fischer’s exact tests were used to compare susceptibility rates. P value < 0.05 was considered statistically significant. This study was approved by the Mayo Clinic institutional review board.

## Results

### Age-stratified antibiogram

Comparison of the cumulative and age-stratified antibiograms is shown in Table 1. For *E. coli,* pediatric isolates were less susceptible to ampicillin and ampicillin-sulbactam and more susceptible to gentamicin and ciprofloxacin compared to adult isolates (p < 0.05 for all). *E. coli* isolates from patients >65 years were less susceptible to ciprofloxacin than were isolates from younger patients (p < .001). There were no differences in susceptibility to trimethoprim-sulfamethoxazole (TMP-SMX) or nitrofurantoin by age.

**Table 1:** **Susceptibility of*E. coli, S. aureus*, and*S. pneumoniae*isolates by patient age, Mayo Clinic Rochester, MN**

	All patients	<18 y	18-64 y	≥65 y	p-value
***E. coli***	n = 3425	n = 310	n = 1465	n = 165	
Ampicillin	55%	48%	55%	56%	0.048
AMP-SLB	48%	40%	48%	50%	0.01
Cefazolin	91%	93%	91%	91%	0.47
Ceftriaxone	96%	96%	96%	98%	0.24
Gentamicin	91%	95%	92%	90%	0.012
Ciprofloxacin	77%	94%	81%	71%	<0.001
TMP-SMX	75%	75%	74%	76%	0.29
Nitrofurantoin	97%	99%	97%	97%	0.18
***S. aureus***	N = 3046	N = 409	N = 1552	N = 108	
Oxacillin or Cefazolin	67%	78%	70%	59%	<0.001
Clindamycin	66%	82%	69%	57%	<0.001
Levofloxacin	68%	90%	73%	52%	<0.001
TMP-SMX	99%	100%	99%	99%	0.054
***S. pneumoniae***	N = 499	N = 139	N = 203	N = 157	
Penicillin (IV)	98%	97%	98%	99%	0.61
Penicillin (oral)	58%	45%	60%	67%	0.001
Ceftriaxone (non-CNS)	98%	96%	98%	99%	0.101
Ceftriaxone (CNS)	87%	74%	92%	92%	<0.001
Tetracycline	73%	60%	81%	74%	<0.001
Erythromycin	52%	37%	60%	54%	<0.001
Levofloxacin	98%	100%	99%	96%	0.019
TMP-SMX	65%	51%	69%	75%	<0.001

Years (y); ampicillin-sulbactam (AMP-SLB), trimethoprim-sulfamethoxazole (TMP-SMX), intravenous (IV), central nervous system (CNS). P-value compares differences across all age groups.

For *S. aureus,* susceptibility to oxacillin, clindamycin, and levofloxacin was highest among children and decreased with increasing age (p < .001 for all). TMP-SMX susceptibility did not change with age. For methicillin-susceptible *S. aureus* (MSSA), clindamycin susceptibility did not vary significantly by age group (84% in children, 79% in adults 18–64 years, and 81% in >65 years). However, for methicillin-resistant *S. aureus* (MRSA), clindamycin susceptibility decreased dramatically with age (76% in children, 43% in adults 18–64 years, 20% in >65 years). For *S. pneumoniae,* pediatric isolates were less susceptible than adult isolates to penicillin (oral breakpoint), ceftriaxone (CNS breakpoint), tetracyclines, macrolides, and TMP-SMX. There were no significant differences in *S. pneumoniae* resistance between the adult age groups.

### Location-stratified antibiogram

Location-stratified antibiograms for *E. coli* and *S. aureus* are shown in Table 2. The institution-wide antibiogram stratified by location, but not age, did not reveal clinically significant differences in susceptibility between IP and OP isolates (data not shown). However, when stratified by both location and age, IP *E. coli* isolates from children and adults 18–64 years were significantly less susceptible to ampicillin, cefazolin, ceftriaxone, gentamicin, and ciprofloxacin than were OP *E. coli* isolates (Table 2). In those >65 years, there were no clinically significant differences between IP and OP *E. coli* susceptibility.

**Table 2:** **Susceptibility of*E. coli, S. aureus*, and*S. pneumoniae*isolates by patient age and location, Mayo Clinic Rochester, MN**

	<18 y			18-64 y			≥65 y		
	IP	OP	p-value	IP	OP	p-value	IP	OP	p-value
***E. coli***	n = 39	n = 267		n = 318	n = 1105		n = 499	n = 1116	
Ampicillin	28%	51%	0.008	48%	57%	0.005	58%	55%	0.19
AMP-SLB	30%	41%	0.19	40%	50%	0.004	52%	50%	0.51
Cefazolin	77%	96%	<0.001	86%	93%	<0.001	90%	92%	0.24
Ceftriaxone	87%	98%	0.004	93%	97%	0.01	96%	98%	0.12
Gentamicin	87%	97%	0.022	89%	93%	0.026	91%	90%	0.64
Ciprofloxacin	82%	96%	0.001	73%	83%	<0.001	73%	71%	0.38
TMP-SMX	59%	77%	0.015	74%	74%	0.91	81%	74%	0.002
Nitrofurantoin	100%	99%	0.99	95%	98%	0.15	97%	97%	0.96
***S. aureus***	n = 91	n = 317		n = 533	n = 957		n = 479	n = 577	
Oxacillin or Cefazolin	77%	78%	0.88	66%	73%	0.003	52%	65%	<0.001
Clindamycin	81%	82%	0.78	62%	73%	<0.001	49%	65%	<0.001
Levofloxacin	88%	91%	0.51	64%	78%	<0.001	45%	58%	<0.001
TMP-SMX	100%	100%	n/a	99%	99%	0.96	99%	98%	0.039

Inpatient (IP), outpatient (OP), ampicillin-sulbactam (AMP-SLB), trimethoprim-sulfamethoxazole (TMP-SMX), oxacillin (ox), cefazolin (cef).

In contrast, for *S. aureus* there were no differences in susceptibility between IP and OP pediatric isolates*,* and small but statistically significant differences between IP and OP adult isolates. For *S. pneumoniae*, there were no significant differences in susceptibility when isolates were stratified by age and location (data not shown).

## Discussion

At our institution age-stratified antibiograms for *E. coli, S. aureus,* and *S. pneumoniae* were significantly different from the institution-wide, cumulative antibiogram. Within age groups, susceptibility varied by IP and OP location at time of specimen collection for *E. coli* but not for *S. aureus* or *S. pneumoniae.*

We observed, as in previous studies, that *E. coli* and *S. aureus* isolates from children were the least drug resistant, while those from patients > 65 years were the most drug resistant (Boggan et al. 2012; David et al. 2006). However, this trend was obscured by the institution-wide antibiogram which reported average values, thus overestimating resistance in pediatric isolates and underestimating resistance in isolates from the elderly. A high prevalence of *E. coli* and *S. aureus* drug resistance in the elderly is well-documented and most likely reflects greater comorbidities, hospitalizations, and antimicrobial exposure among older patients (David et al. 2006; Swami et al. 2012). In contrast, for *S. pneumoniae*, pediatric isolates were more drug resistant than adult isolates. The greater drug resistance among pediatric *S.pneumoniae* isolates is probably due to the high utilization of penicillins in children and the likelihood that the *S. pneumoniae* isolates used to create the antibiogram reflected complicated or refractory infections, since *S. pneumoniae* is not routinely cultured in uncomplicated otitis media or pneumonia.

Clinician reliance on institution-wide antibiograms that do not accurately reflect susceptibility rates in certain patient groups might lead to inappropriate empiric antibiotic prescribing. Overestimating resistance in pediatric *S. aureus* or *E. coli* could lead to prescribing of unnecessarily broad antibiotics in children which, in turn, can lead to increasingly drug resistant pathogens and *C. difficile* infections. For example, we have observed that local providers avoid clindamycin for treatment of pediatric skin and soft tissue infections because the cumulative antibiogram reports high rates of clindamycin resistance. An age-stratified analysis revealed that in our geographic region, clindamycin susceptibility among pediatric *S. aureus* isolates is above 80% and this drug remains an important therapeutic option for treatment of skin and soft tissue infections, including MRSA, in children but not in older adults. Similarly, providers in our community have followed national trends (Copp & Hersh 2011) by increasingly prescribing third generation cephalosporins for empiric treatment of pediatric urinary tract infections. Prescribing of fewer broad - spectrum agents could be encouraged by creation and dissemination of a pediatric antibiogram demonstrating *E. coli* with high susceptibility to narrow - spectrum cephalosporins. Boggan et al. recently reported that effective antibiotic prescribing among pediatricians, as measured through responses to clinical vignettes, improved when pediatric-specific antibiograms were provided (Boggan et al. 2012).

For elderly patients, availability of age-specific antibiograms that do not underestimate drug resistance is also likely to enhance appropriate antibiotic selection, which in turn, can optimize outcomes (Ibrahim et al. 2000). We recently determined that in our county, a quarter of elderly patients with fluoroquinolone-resistant *E. coli* infections received ineffective empiric therapy with a fluoroquinolone and had persistent or recurrent infections, likely due to lack of awareness among providers about local resistance rates in this age group. Furthermore, because residents of long-term care facilities are at high risk for colonization or infection with multidrug-resistant organisms, some have advocated creation of antibiograms specific for long-term care facility residents (Philippe et al. 2011). However, because many long-term care facilities lack resources to create their own antibiograms, a more practical option may be for providers to rely on hospital-based age-stratified antibiograms, as we have created.

Within age groups, we noted clinically significant differences in susceptibility of IP and OP isolates of *E. coli* but not of *S. aureus* or S*. pneumoniae*. *E. coli* OP isolates were generally more drug susceptible than IP isolates, especially among children. In contrast, for *S. aureus*, differences in susceptibilities between IP and OP isolates from children as well as adults were not statistically significant. These findings suggest that age- and location- stratification of *E. coli*, the most common pathogen isolated in children and in urinary tract infections in general, might be a valuable tool to guide empiric antibiotic selection for management of pediatric urinary tract infections.

This study has several limitations. Since it was laboratory-based, we did not have associated clinical history and could not determine if cultures represented infection or colonization, or were community-associated vs. healthcare-associated isolates. Although we eliminated duplicate isolates, our sample was also likely biased toward patients with complicated or refractory infections and prior antibiotic exposure, since such patients have cultures sent more frequently than patients with uncomplicated infections. Thus, the isolates in our collection may be more drug-resistant than those in the general population. Lastly, our susceptibility data reflect local epidemiology and may not be generalizable to other geographic regions or institutions. Despite these limitations, we demonstrate that creation of age and location-stratified antibiograms is feasible and valuable.

In conclusion, stratified antibiograms reveal age - associated differences in susceptibility of *E. coli, S. aureus*, and *S. pneumoniae* that are obscured by hospital-wide antibiograms. Further stratification of *E. coli* isolates by both age and IP or OP location may also be useful to clinicians who manage pediatric urinary tract infections. Although the proportion of institutions that create stratified vs. cumulative antibiograms is not clear, we believe that more facilities should create age - stratified antibiograms especially if they serve diverse patient groups (i.e. are not free-standing children’s hospitals or long-term care facilities). More research is needed to determine if improved antibiograms can be valuable stewardship tools that facilitate appropriate empiric antibiotic selection and enhance surveillance of antimicrobial resistance trends (Hebert et al. 2012; McGregor et al. 2009).

Previously presented in abstract form at the Infectious Disease Society of America meeting, 2011, Boston, MA.
